# Feasibility of Using a GENEActiv Accelerometer with Triaxial Acceleration and Temperature Sensors to Monitor Adherence to Shoulder Sling Wear Following Surgery

**DOI:** 10.3390/s24030880

**Published:** 2024-01-29

**Authors:** Ahmed Barakat, Abdurrahmaan Manga, Aneesa Sheikh, Ryan McWilliams, Alex V. Rowlands, Harvinder Singh

**Affiliations:** 1 Trauma & Orthopaedics Department, University Hospitals of Leicester NHS Trust, Leicester LE5 4PW, UK; ahmedharoonbarakat@gmail.com (A.B.);; 2 Assessment of Movement Behaviours (AMBer), Leicester Lifestyle and Health Research Group, Diabetes Research Centre, University of Leicester, Leicester LE5 4PW, UK; 3 National Institute for Health Research, Leicester Biomedical Research Centre, Leicester LE5 4PW, UK; 4 Alliance for Research in Exercise, Nutrition and Activity (ARENA), UniSA Allied Health and Human Performance, Division of Health Sciences, University of South Australia, Adelaide 5000, Australia; 5 School of Healthcare, University of Leicester, Leicester LE1 7RH, UK

**Keywords:** shoulder, sling, compliance, accelerometer, IMU, treatment, fidelity, surgery

## Abstract

Background: Self-reported adherence to sling wear is unreliable due to recall bias. We aim to assess the feasibility and accuracy of quantifying sling wear and non-wear utilising slings pre-fitted with a GENEActiv accelerometer that houses triaxial acceleration and temperature sensors. Methods: Ten participants were asked to wear slings for 480 min (8 h) incorporating 180 min of non-wear time in durations varying from 5–120 min. GENEActiv devices were fitted in sutured inner sling pockets and participants logged sling donning and doffing times. An algorithm based on variability in acceleration in three axes and temperature change was developed to identify sling wear and non-wear and compared to participants’ logs. Results: There was no significant difference between algorithm detected non-wear duration (mean ± standard deviation = 172.0 ± 6.8 min/participant) and actual non-wear (179.7 ± 1.0 min/participant). Minute-by-minute agreement of sensor-detected wear and non-wear with participant reported wear was 97.3 ± 1.5% (range = 93.9–99.0), with mean sensitivity 94.3 ± 3.5% (range = 86.1–98.3) and specificity 99.1 ± 0.8% (range = 93.7–100). Conclusion: An algorithm based on accelerometer-assessed acceleration and temperature can accurately identify shoulder sling wear/non-wear times. This method may have potential for assessing whether sling wear adherence after shoulder surgeries have any bearing on patient functional outcomes.

## 1. Introduction

Shoulder sling immobilisation after shoulder surgery, such as rotator cuff repairs or shoulder arthroplasties, is usually advocated as an integral part of the post-operative rehabilitation [1]. Elucidating the ideal sling immobilisation protocol is important as it is postulated that inadequate treatment can lead to higher rates of retears while over treatment would eventually lead to a frozen shoulder, with both culminating in suboptimal functional outcomes. For that reason, optimal duration of sling wear is a matter of debate with conflicting evidence supporting a wide range of recommended durations.

For example, a systematic review by Baumgarten et al. could not reach a recommendation for post rotator cuff repair rehabilitation or protection protocols based on the available level I and II evidence [2]. A more recent systematic review of overlapping meta-analyses by Houck et al. reaffirmed the challenge in ascertaining the fine balance for the optimal immobilisation duration as it showed that early rehabilitation as opposed to temporary sling immobilisation leads to better range of motion but on the expense of higher rate of retears [3]. Contrarily, another synchronously published systematic review by Saltzman et al. concluded that the best available evidence demonstrates similar functional outcomes and retear rates when comparing early versus delayed rehabilitation protocols following rotator cuff repairs [4]. Mazzocca et al. prospectively randomised 73 patients to early and delayed rehabilitation groups with no demonstrated difference in functional scores or radiologically assessed retear rates after six months [5]. Jensen et al. randomised 120 patients to a simple sling for 3 weeks versus a brace with a small abduction pillow with the arm in neutral position for six weeks after small- to medium-sized rotator cuff tear repairs. They reported non-inferiority of the three-week regimen when compared to the 6-week regimen in terms of functional scores and retear rates [6]. A later prospective randomised controlled trial by Tirefort et al. randomised 80 patients to two matched sling and no-sling cohorts following arthroscopic repair of small and medium sized rotator cuff tears. After 6 months follow-up duration, it was found that the no-sling cohort had better range of motion, functional scores and without statistically significant retear rates as confirmed by ultrasonography [7]. In all those studies, adherence was self-reported and not validated either by self-reported questionnaires or more objectively by sensor-based methods. In the context of this uncertainty, current practice is to follow a generally cautious approach, including long periods of immobilisation in a sling and avoidance of early active rehabilitation.

We believe that it is the patient’s adherence (i.e., the percentage of actual wear duration relative to the prescribed wear duration) to the recommended treatment is what trumps the standardised recommended treatment duration. Even with the available high quality evidence studies which were prospective randomised controlled trials, sling adherence is still poorly alluded to. In these trials, even matching of different cohorts in terms of patient and surgical factors, standardisation of post-operative rehabilitation and immobilisation protocols still leaves the unsupervised patient’s adherence to sling immobilisation unaccounted for and a highly plausible confounding factor.

The term adherence itself is subtly different from the term compliance and this difference warranted us to prefer the former for our study. Compliance implies passive following of the patient to the prescriber care plan while the more encompassing adherence term reflects a more pro-active patients’ involvement in the care plan decision and the extent to which they match this mutually decided plan. Non-compliance was regarded as synonymous with patient’s disobedience to the treatment plan which unjustly and solely blamed the patient. Contrarily, patient’s non-adherence can be due to non-feasible treatment plans such as un-necessarily prolonged immobilisation, patient’s inadvertent forgetfulness or cognitive lack of capacity [8].

It has already been shown that poor adherence to treatment (sling wear in this case as an integral part of post-operative rehabilitation) leads to unsatisfactory outcomes and even adverse effects which adds the toll of the medical expenditure [9]. As it turns out, the theme of treatment non-adherence is consistent among different modes of orthopaedic bracing. This further emphasizes the diminished value of entrusting the treatment duration (quantity) as an effective mean of standardisation and avoiding confounding bias without a notion to the treatment adherence (quality) itself. A systematic review collated 19 studies concerned with treatment adherence to scoliosis, clubfoot, ankle, and knee immobilizer braces unanimously found inadequate adherence to all of them which ranged from 27% to 85% at best [10].

Assessing treatment adherence has been previously described either subjectively by means of patient questionnaires and more recently objectively validated by more robust monitoring adjuncts. Undeniably, assessing adherence using patient questionnaires conceals an extent of intrinsic subjectivity. Understandably, there is always a room for recall bias giving falsely exaggerated adherence rates. This has been shown by studies comparing patient self-questionnaires finding lower actual treatment adherence when cross referenced with sensor monitored treatment adherence [11,12]. Wearable accelerometers have been rigorously validated in a recent systematic review on treatment adherence studies validating their accuracy in capturing and quantifying upper limb movements [13]. Given this, wearable accelerometers have potential for capture of the movements associated with sling wear and the actions of sling doffing and donning. However, this may be confounded by accelerations recorded if the sling is removed, but still moved, e.g., if the person carries the sling around, e.g., in a bag. In this case, considering accelerations alone will erroneously classify the sling as being worn.

Objective assessment of sling treatment adherence has been previously attempted with temperature sensors incorporated in a sling to determine whether the sling is in contact with the body, i.e., is being worn [14]. Understandably, temperature change is gradual thus there was a time lag between temperature measured and the actual sling donning or doffing potentially impacting on the accuracy of monitoring of adherence to treatment [15]. Further, studies using only temperature sensors for assessment of sling wear adherence require continuous body contact with the implanted sensors as temperature fluctuations can occur if the sensor is not flush against the body [14]. Combining temperature and acceleration measures may mitigate the weaknesses associated with either approach alone, thus optimising monitoring of sling adherence. Triaxial accelerometers are relatively small and low weight sensors which can accurately capture both multi-planar acceleration motion and temperature [16]. Despite having ample evidence validating their accuracy for biomonitoring, to our knowledge, their use has not been attempted to date in the context of sling wear adherence [17].

**Figure 1:**
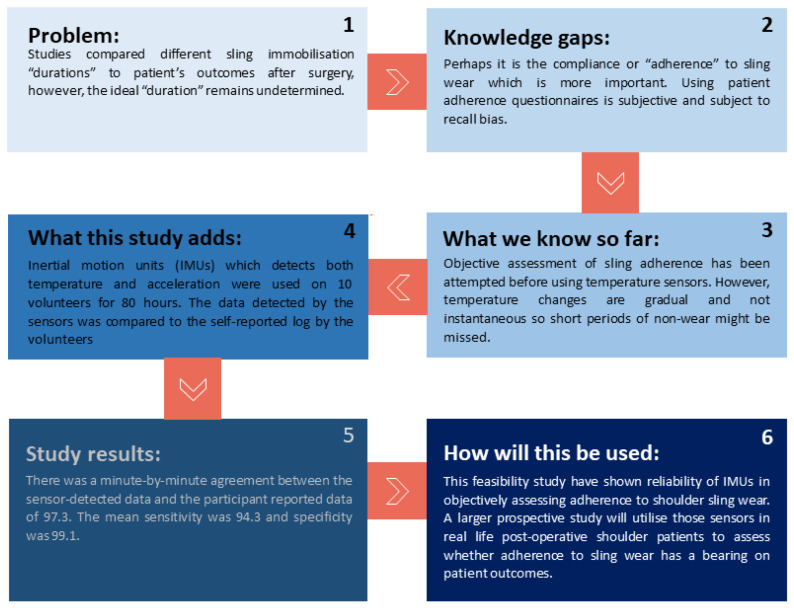
Research roadmap depicting the background for the study, design, and future use case.

In this study, we evaluated the accuracy of an algorithm for detection of sling wear/non-wear from a GENEActiv accelerometer (that houses triaxial acceleration and temperature sensors) secured in the shoulder sling. Development of an accurate objective measure of sling wear adherence would pave the way for future studies concerned with understanding of patient/surgical factors affecting adherence to sling wear (Figure 1).

## 2. Materials and Methods

Ten volunteers (volunteers applied through Leicester University Medical Research Society (LUMRS) platform where recruitment initiatives for similar studies and trials are advertised) provided written informed consent prior to data collection. This study received ethical approval from the National Research Ethics Committee (REC) Integrated Research Application System (IRAS, reference: 315132).

**Figure 2:**
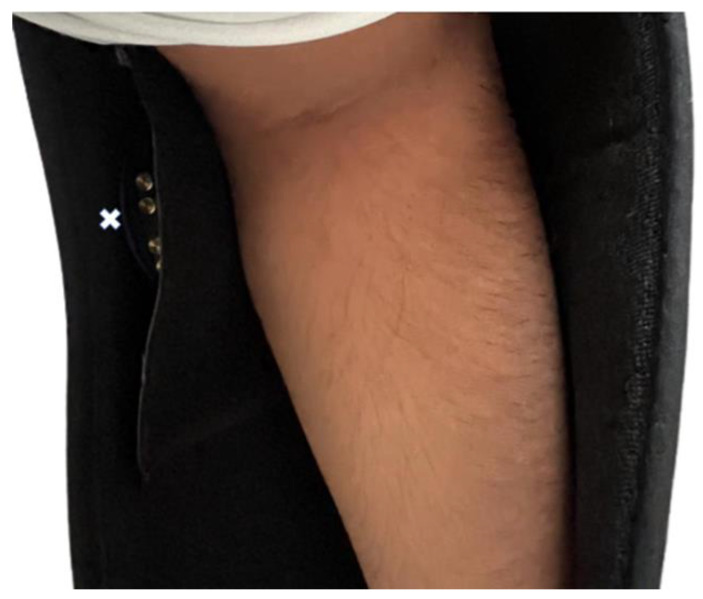
Position of the GENEActiv (marked with a white x symbol) fitted in an inner sling pocket and next to the participant’s arm.

Participants were asked to wear a universal shoulder immobilisation sling in which the GENEActiv accelerometer was secured (GeneActiv, Activinsights, Cambridgeshire, UK) (Figure 2). Participants were asked to go about their normal daily activity, removing the sling for scheduled periods of non-wear.

**Figure 3:**
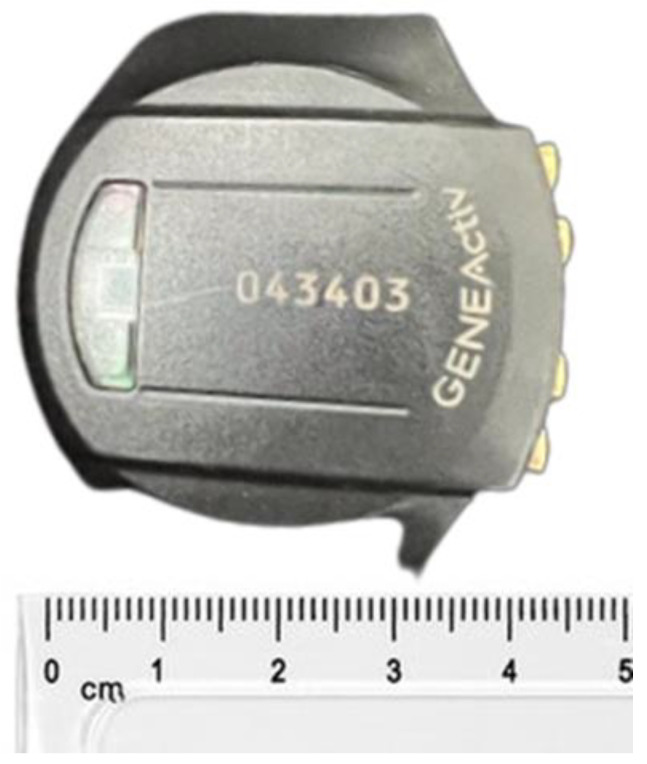
GENEActiv accelerometer (housing triaxial acceleration and temperature sensors).

The GENEActiv weighs 16 g and measures 43 × 40 × 13 mm in dimensions (Figure 3). It contains a MEMS sensor with a dynamic range of ±8 *g* and 12 bit (3.9 mg) resolution and a linear active thermistor with a range of 0–60 °C, resolution of 0.25 °C. The ultra-low battery consumption of these sensors allows collection of acceleration in three axes at 10 Hz for up to 45 days on a single battery charge; at the maximum measurement frequency of 100 Hz used in this study, the measurement period is seven days on a single charge.

The triaxial accelerometers were initialised in GENEActiv PC (version 3.3) to collect data at 100 Hz. Each participant was asked to wear the pre-fitted sling for a 480-min (eight-hour) period containing a series of 180-min of non-wear periods in pre-specified durations varying from 5- to 60-min. Data were downloaded, and 60-s epoch comma separated values (CSV) files generated in GENEActiv PC. Thus, there were up to 480 60-s epochs per participant. Minimum sample size for sensitivity studies ranges between 60 to 4860 [18]. Our study is at the top end of this range for similar studies and was sufficiently powered with >4800 measurements logged across all participants.

Progressive non-wear times were implemented (from five-minutes to 120 min) to determine the impact of non-wear duration on detection. The order of the non-wear durations was randomised for each participant using computer generated randomisation. Sling wearers were asked to log sling wear, donning, doffing and wear times to accurately cross-reference with accelerometer recorded data. At the termination of the eight hours sling wear, the logged data was downloaded to a computer using a universal serial bus (USB) cable.

Although self-reported adherence has not proven reliability in previous adherence assessment studies, these studies were relying on assessment of a patient cohort who were liable to both recall bias and Hawthorne effect both unintentionally falsely exaggerating self-reported adherence rates [3,4]. This was avoided in our study by asking healthy volunteers to follow a pre-determined non-wear schedule and immediately log the exact donning and doffing times in a logbook to avoid any late recall bias. Moreover, volunteers understood the aim of this study and were briefed with a volunteer specific sling wear and non-wear schedule as previously mentioned.

### 2.1. Sling Non-Wear Algorithm

All ten participants were asked to wear the pre-fitted slings for 480 min with 180 min of non-wear embedded into their wear schedule at randomised intervals different for each participant. These non-wear periods ranged from 5- to 120-min (5, 10, 15, 30, and 60–120 min). The participants were asked to accurately log the donning and doffing times to the minute using watches synchronised with an internet clock (https://time.is, accessed 1 May 2022).

GENEActiv acceleration and temperature data were imported into a custom-built Excel sheet for detection of non-wear. To identify periods of non-wear, we employed a rolling three-minute average based on minute-by-minute temperature changes and a rolling two-minute average in the standard deviation (SD) of the acceleration. The start of non-wear was defined as a moment when this rolling three-minute average of temperature change dropped below −0.2 °C and concurrently, a rolling two-minute average of the SD of all three of the axes of acceleration fell below 13 mg. Conversely, the end of non-wear was marked by a subsequent increase in the rolling three-minute average temperature change exceeding 0.1 °C and a rolling two-minute average of the SD of any of the three axes rising above 13 mg. These thresholds were based on those used to detect non-wear of the wrist-worn GENEActiv in the macro developed by ActivInsights (Cambridgeshire, UK, https://activinsights.com/) [19].

Different time constants were used for temperature and acceleration as temperature changes occur more slowly compared to acceleration. Thus, a shorter time constant for acceleration is necessary to capture quick detection of device removal or non-wear, while a longer accounts for the slower changes that are evident with temperature. This approach allows us to identify non-wear periods in a manner that accounts for short-term fluctuations while maintaining a continuous assessment of the data [11].

### 2.2. Analyses

Minute-by-minute participant-logged data (determined from donning and doffing times) were timestamp matched to minute-by-minute wear/non-wear detected by our algorithm. Sensor-determined and self-reported total non-wear were compared using paired *t*-tests; intraindividual classification agreement across 60-s epochs was reported as percent agreement, sensitivity, specificity, and Cohen’s kappa [20]. All analyses were conducted using SPSS V.25 (IBM, Armonk, NY, USA).

## 3. Results

**Figure 4:**
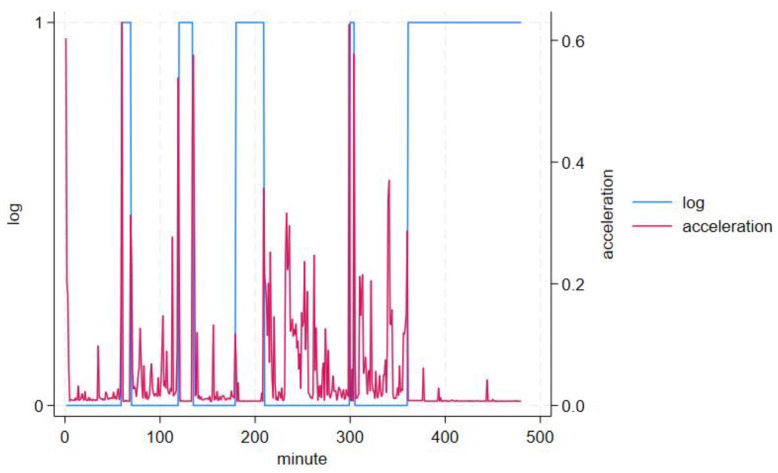
Standard deviation of acceleration (*g*, in one acceleration axis only for clarity, red) and self-reported non-wear (log, blue); 1 is non-wear while 0 is wear on the left Y-axis.

**Figure 5:**
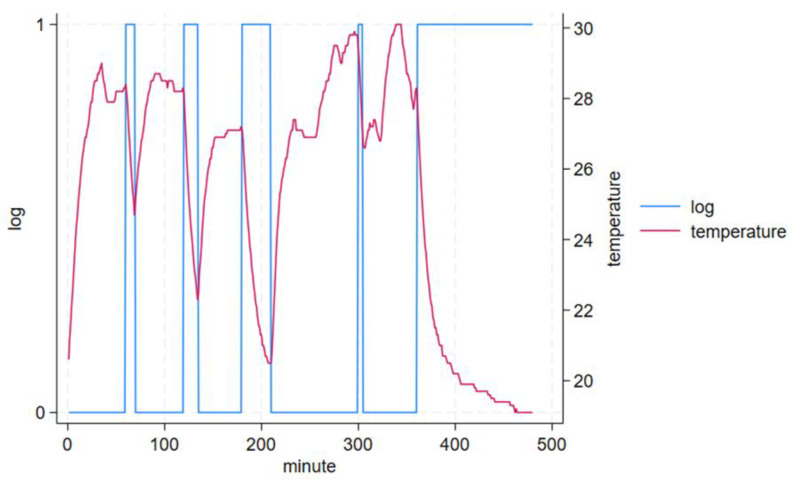
Temperature (red) as recorded by the sensor-fitted slings (blue line) versus self-reported non-wear (log, blue); 1 is non-wear while 0 is wear on the left Y-axis.

**Figure 6:**
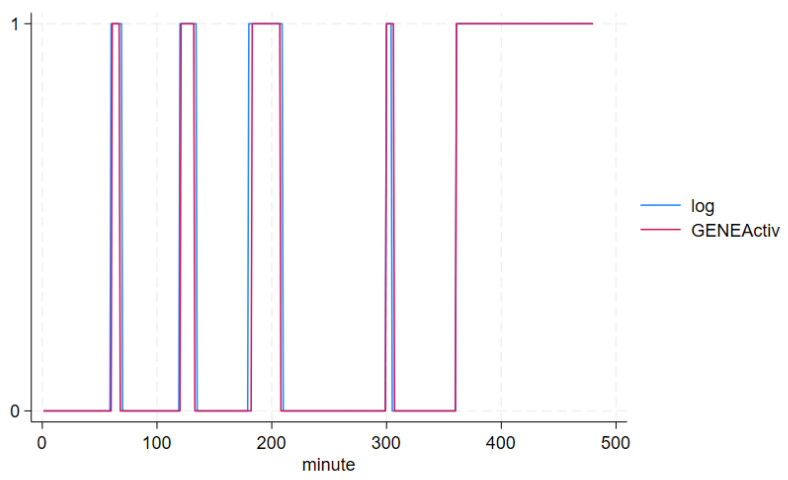
Algorithm determined (GENEActiv, red) and self-reported (log, blue) non-wear (0 on Y-axis) and wear (1 on Y-axis).

Ten volunteers participated in our study. Their mean age was 28.5 years (range = 20–57 years, SD = 16.6), five were males (50%) and five were females (50%). The study duration was 480 min per participant, including ~180 min non-wear. A total of 179.7 ± 1.0 min (range = 177–180 min) non-wear across a mean of 4.8 ± 0.4 (range = 4–5) non-wear occurrences lasting 5–120 min each were reported by participants. Mean epoch-by-epoch acceleration (signal vector magnitude, SVM) and temperature by logged wear and non-wear periods are shown in Table 1. GENEActiv-detected changes in acceleration (Figure 4), temperature (Figure 5) and algorithm determined non-wear (Figure 6) closely tracked participant recorded wear/non-wear times; data shown for a representative participant.

The algorithm detected a total of 172.0 ± 6.8 min (range = 159–193 min) non-wear across a mean of 4.8 ± 0.4 (range = 4–5) non-wear occurrences. Sensor-determined and self-reported total non-wear was compared using a paired *t*-test with no statistical difference found (*p* = 0.14). Mean epoch by epoch (minute-by-minute) agreement of wear/non wear was 97.3 ± 1.5% (Range = 85.8–99.0). The mean sensitivity for all 10 participant’s data was 94.3 ± 3.6% (range = 92.8–98.3) and mean specificity was 99.1 ± 0.8% (range = 93.7–100). Reliability between logged data and measured data for each participant was assessed using Kappa co-efficient for inter-measurement agreements and was a mean of 0.94 ±0.03 (Range 0.89–0.98) which translates to ‘almost perfect’ agreement and detection of non-wear times [20].

All non-wear occurrences were detected, even as short as five minutes, in all participants. Table 2 shows the actual and detected non-wear duration for the scheduled non-wear durations (5, 10, 15, 30 min and longer durations of 60–120 min). Of note, the duration of the shorter (5–10 min) non-wear periods were overestimated by the algorithm when benchmarked against the participant logged non-wear times. This was at most 17.5% overestimation for the shortest non-wear occurrence of five minutes which represents the potential lag in measurements logged by these accelerometers.

## 4. Discussion

Adherence to sling wear was determined with 97% accuracy using an algorithm applied to acceleration and temperature data from an accelerometer embedded in a sling worn by healthy participants. All non-wear occurrences were identified with a high concordance between participant self-reported donning and doffing times and sensor recorded data. During short non-wear periods, the algorithm tended to over-estimate the duration of non-wear, with the greatest overestimation being 17.5% for 5 min of non-wear. However, there was no over-estimation in 50% of the participants (*n* = 4), 1-min overestimation on 25% of participants (N = 2), and 2–3 min overestimation in the remaining 25% (N = 2). It is possible that this overestimation may be due to small logging errors, or due to an asymmetry in temperature lag in some participants when putting the sling on and removing it. This may occur particularly where environmental conditions are different between donning and doffing times. However, we believe that this difference (range: 0 to 3 min overestimation) would not have a clinically significant impact on the accuracy of detecting non-wear periods.

Assessment of sling wear adherence has been attempted before using temperature sensors. Sood et al. cross-referenced self-recorded sling wear in four participants with data logged from temperature sensors fitted to the slings to develop an algorithm for validating actual wear adherence [15]. They used three temperature sensors at different locations in the sling closely adherent to the volunteer’s body. Their algorithm modelled sling donning/doffing times by analysing cutoff temperatures and they found a diagnostic accuracy >99% for the three locations. A large-scale study conducted by Grubhofer et al. on 54 shoulder abduction braces fitted with temperature sensors for post-operative rotator cuff repair patients. The authors reported that self-reported adherence was significantly less than the sensor-measured adherence and concluded self-reported adherence is unreliable [21]. Another prospective study by Livesey et al. assessed sling wear adherence in 66 post-operative shoulder patients for one month after their procedures using similar temperature sensors [22]. The study aimed to evaluate the associations between postoperative sling wear adherence and patients’ understanding of sling necessity, postoperative home assistance, and social deprivation. The authors, similarly to previous studies, found that patients with greater understanding for sling necessity, those with home assistance, and patients >60 years have greater adherence to sling wear, while male patients and those with a higher BMI had lower sling adherence rates. Despite the reported high accuracy [15] for these sensors in capturing actual wear time, the temperature was recorded at 15-min intervals and the authors submitted that this may potentially underestimate the wearing time by about 30 min per wearing session. Moreover, the accuracy of this method can be potentially limited by different environmental conditions and/or wearing the sling, then leaving it in a warm environment. Finally, the time lag between temperature measured and actual sling donning or doffing potentially impacts on the accuracy of monitoring of adherence to treatment. This is mitigated in the current study by a dual sensor approach where the combination of acceleration and temperature mitigates the weaknesses of either method used alone. This method was able to detect all non-wear occurrences in all participants including those as short as 5 min. It is worth noting that in those studies objectively validating actual sling wear using temperature sensors with reported high accuracy, no attempt was made to assess the effect of different validated adherence rates on patient outcomes.

The relationship between self-reported sling wear adherence and functional outcomes after shoulder surgery has been previously investigated. Silverio and Cheung assessed 50 consecutive patients who had rotator cuff repairs [23]. Patients were instructed to wear an abduction brace for six weeks after surgery. Different functional evaluations, including American Shoulder and Elbow Surgeons score (ASES), University of California-Los Angeles shoulder score (UCLA), and Simple Shoulder Test, were performed preoperatively and after their surgical procedures. The average adherence as assessed by patient self-reported questionnaires was 88% with no significant correlations between adherence rates and improvement in any of the three different outcomes measures assessed. The authors concluded that adherence rates do not impact patient’s outcomes after rotator cuff surgeries. However, adherence was only subjectively assessed by patient reported medical adherence measurement questionnaires, and as shown previously, there is a disconnect between self-reported adherence and objective sensor-based adherence, with the latter being significantly lower. We are proposing a novel inertial sensor-based validation of sling wear adherence which we will incorporate in a subsequent prospective study to assess the effect of this validated adherence on patient functional outcomes using the GENEActiv accelerometers.

The GENEActiv accelerometers are low weight sensors (around 20 g each) with a battery life that can last for a six-week immobilisation period without the need to be charged or any intervention from the patient. This makes them ideal for unimpeded monitoring throughout the treatment course. Detection of acceleration data in the three axes as well as temperature is advantageous for detection of sling non-wear. Studies using temperature sensors alone show continuous body contact is required with the implanted sensors to decrease fluctuations that can occur if the sensor is not flush against the body. Additionally, there is a time lag between temperature measured and the actual sling donning or doffing potentially impacting on the accuracy of monitoring of adherence to treatment. This is mitigated when using acceleration in addition to body temperature, although continuous body contact would still be required with our algorithm.

Accelerometers have been previously used with comparable high accuracy in assessing shoulder kinematics and range of motion for investigative purposes. They have been rigorously validated in a recent systematic review validating their accuracy in capturing and quantifying upper limb movements [8]. Ruder et al. has used similar accelerometers for assessment of shoulder movements in post-rotator cuff tear repair patients against control subjects and reported high accuracy and precision for shoulder movement monitoring [24]. Their preliminary data demonstrated the ability to discriminate between healthy control subjects and patients recovering from rotator cuff repair and provide support for using a wearable wrist sensor to monitor changes over time in shoulder activity. Further, Parel et al. used accelerometers for assessment and validation of ambulatory scapulothoracic rhythm in 40 subjects [25]. Their study aimed to evaluate the feasibility of using a single IMU to recognise and classify a supervised shoulder rehabilitation activity. The authors demonstrated that these sensors performed well with a high accuracy and validity in detecting and categorising those rehabilitation activities. Kaszynski et al. evaluated the reliability of the RSQ Motion (RSQ Technologies, Poznan, Poland) sensor and its concurrent validity in measuring the active range of motion of the shoulder in 15 healthy individuals and likewise, demonstrated high validity and reliability of these IMUs in the context of shoulder ROM assessment [26]. These studies demonstrate the feasibility of using accelerometers in a rehabilitation setting and thus their potential for also being used to assess adherence to sling wear.

Broadly, an additional advantage of monitored adherence is that goal setting and gamification could potentially be added to rehabilitation programmes to bolster patient’s treatment adherence. Akin to wearables such as health trackers and pedometers, treatment adherence monitors may be able to positively influence behaviour and motivate stricter adherence [27]. The mere knowledge that the treatment is being monitored may lead to better adherence rates as shown in a prospective study demonstrating a statistically significant 29% increase in adherence rates when patients were aware that the treatment was being monitored [27]. Moreover, monitored treatment adherence can provide clinicians with more insight about the progress of the patient’s rehabilitation and prompts them to intervene early if warranted.

In clinical practice it would only be feasible to download data at pre-determined time-points during clinical encounters. In this study, we used a 100 Hz measurement frequency which limited the data storage to seven days. In a true patient scenario, setting the measurement frequency to 10–20 Hz would extend storage and battery life to at least 1–2 months making it more clinically practical without the need to download logged data before that time. Although the magnitude of acceleration may be reduced at lower sampling frequencies, there appears to be minimal impact on the variability of accelerations (suggesting this algorithm would perform similarly on data collected at lower sampling frequencies [28]. However, this should be confirmed in further research. Other commercially currently available accelerometers can deliver real-time data sent wirelessly to a secure Internet-based data cloud for up to six months battery life [29].

Real-time monitoring and access to the measured data has the potential to be a true game changer for patients’ management plans. For example, treatment plans and rehabilitation could be patient specific and adjusted according to their degree of adherence to treatment and pattern of non-adherence. Only mild adjustments may be needed for patients with mild non-adherence whereas more drastic adjustments and interventions may be warranted for patients with increasingly inadequate adherence. Moreover, the continuous monitoring capabilities of accelerometers would contribute to a more dynamic understanding of patient behaviour over time. Patterns of adherence and non-adherence and the degree to which adherence may be associated with patient demographics, health status, the nature of their shoulder condition and shoulder function could be identified.

Taking it further from other related validation studies, we intend to assess the impact of this validated sling wear adherence on the patient functional outcomes after rotator cuff tears which has not been attempted before. This will be the first time to explore an alternate feasible sensor-based validation of sling wear adherence using accelerometers. As previously stated, our method avoids the downfall of temperature sensor-based validation studies such as the time lag and the time dependent measurement rather than the event dependent measurement which correlates more adherently to the donning and doffing events.

## 5. Conclusions

To the extent of our knowledge, this is the first attempt to explore an alternate feasible dual-sensor-based validation of sling wear adherence using triaxial accelerometers. Our algorithm demonstrated 97% accuracy of these accelerometers in defining sling wear times including non-wear periods as short as 5 min. By combining acceleration and temperature the algorithm mitigates the weaknesses of either approach alone, e.g., the time lag for the body temperature to be detected or dissipated by the sensors after donning or doffing, respectively, or continued movement due to a sling being carried in a bag. Further research is needed to test the feasibility and validity of this methods of monitoring sling adherence in a larger more diverse sample size but most importantly in patients. If feasible and valid, it would then be possible to implement in studies assessing sling-wear adherence patterns. The GENEActiv device used incorporates triaxial acceleration and temperature sensors; the algorithm presented herein may be applicable to other research-grade accelerometers that contain triaxial acceleration and temperature sensors, but this would need to be tested. Further, it may be beneficial to consider whether flexible wearable sensors are a feasible alternative [30].

This accelerometer-based monitoring of sling wear adherence can facilitate the way for future studies aiming to define sling wear recommendations more accurately by relating adherence rates to patient outcomes. This will avoid over or under treatment durations which can lead to either stiffness or compromise of surgical outcomes, respectively. Additionally, adjusting treatment plans and rehabilitation will be more patient specific according to their degree of adherence to treatment and pattern of non-adherence, where only mild adjustments are needed for mild non-adherence whereas more drastic adjustments and interventions are warranted for increasingly inadequate adherence.

## Figures and Tables

**Table 1:** Mean ± SD acceleration and temperature during sling wear and non-wear.

	Wear	Non-Wear
Acceleration (SVM) (*g* · min)	161.3 ± 69.9	88.6 ± 24.4
Temperature (°C)	29.1 ± 1.5	24.4 ± 1.8

SVM: Signal vector magnitude.

**Table 2:** Mean measured non-wear duration by duration of non-wear period.

Logged Non-Wear Duration in Minute, (Number of Occurrences)	Measured Non-Wear Duration (Minutes, Mean ± SD)	Percentage Duration of Actual Non-Wear Measured (Mean ± SD)
5 (N = 8)	5.9 ± 1.1	117.5 ± 22.5
10 (N = 8)	10.3 ± 2.8	102.9 ± 27.5
15 (N = 8)	12.9 ± 1.3	85.8 ± 8.3
30 (N = 6)	26.0 ± 3.9	86.7 ± 13.1
60–120 (N = 18)	74.6 ± 18.9	96.6 ± 4.1

N = number of non-wear periods considered for each duration.

## Data Availability

The data that support the findings of this study are not openly available due to containing information that could compromise research participant privacy/consent. Requests for participant-level quantitative data and statistical codes should be made to the corresponding author. Data requests will be put forward to members of the original trial management team who will release data on a case-by-case basis.
